# Custodiol-N Is Superior to Custodiol^®^ Solution in Experimental Rat Uterus Preservation

**DOI:** 10.3390/ijms21218015

**Published:** 2020-10-28

**Authors:** Viktorija Zitkute, Mindaugas Kvietkauskas, Vygante Maskoliunaite, Bettina Leber, Diana Ramasauskaite, Kestutis Strupas, Philipp Stiegler, Peter Schemmer

**Affiliations:** 1 General, Visceral and Transplant Surgery, Department of Surgery, Medical University of Graz, Auenbruggerplatz 2, 8036 Graz, Austria; viktorijazitkute@gmail.com (V.Z.); min.kvietkauskas@gmail.com (M.K.); bettina.leber@medunigraz.at (B.L.); peter.schemmer@medunigraz.at (P.S.); 2 Faculty of Medicine, Vilnius University, M. K. Ciurlionio 21, 03101 Vilnius, Lithuania; vygantem@gmail.com (V.M.); diana.ramasauskaite@santa.lt (D.R.); kestutis.strupas@santa.lt (K.S.); 3 National Center of Pathology, Affiliate of Vilnius University Hospital Santaros Klinikos, P. Baublio 5, 08406 Vilnius, Lithuania

**Keywords:** uterus transplantation, infertility, ischemia reperfusion injury, preservation, static cold storage

## Abstract

Uterus transplantation (UTx) is the first and only available treatment for women with absolute uterine factor infertility. However, clinical application is limited by the lack of organs, ischemia/reperfusion injury, as well as immunosuppression after UTx. Several different preservation solutions are used in experimental and clinical UTx, including Custodiol^®^ solution. Recently, the novel Custodiol-N solution was developed with superior results in organ preservation. However, the solution was not tested yet in UTx. Therefore, the aims of this study were to evaluate the effect of Custodiol-N in uterus prolonged cold preservation time (8 and 24 h), compared to Custodiol^®^ solution. Uterus tissue samples were obtained from adult Sprague Dawley rats (*n* = 10/group). Cold ischemic injury was estimated by histology, including immunohistochemistry, and biochemical tissue analyses. After 8 h of cold ischemia, higher percentage of tissue edema, necrosis signs and myeloperoxidase expression, as well as lower superoxide dismutase activity were found in Custodiol^®^ compared to Custodiol-N (*p* < 0.05). These differences were more pronounced after 24 h of cold preservation time (*p* < 0.05). This study demonstrated that Custodiol-N protects uterus grafts from cold ischemic injury better than standard Custodiol^®^ most likely via inhibition of oxidative stress and tissue edema. It seems that iron chelators in the composition of Custodiol-N play an important protective role against cold ischemia.

## 1. Introduction

Uterus transplantation (UTx) is the first and only available treatment for absolute uterine factor infertility (AUFI). Up to 7% of women suffer from AUFI [1,2], which is linked to either congenital uterine agenesis (Mayer–Rokitansky–Küster–Hauser syndrome), major congenital uterine malformation (hypoplastic uterus, fraction of bicornuate/unicornuate uterus), a surgically absent uterus, or an acquired condition (intrauterine adhesions, leiomyoma) related to uterine malfunction that causes implantation failure or defect placentation [3].

In 2014, the first birth of a healthy child to a woman who underwent UTx under the care of Brännström’s team in Sweden finally removed the doubts and skepticism in the medical community [4,5]. Since then, several births have occurred in multiple centers worldwide utilizing uterus grafts from both living and deceased donors [6]. However, wider clinical application is inherently limited by an organ shortage, ischemia/reperfusion injury (IRI), since all of these factors limit success rates of UTx [2]. To date, the living donor is preferred in UTx, [7] due to the possibility of better donor evaluation and elective planning of the operation. On the other hand, the use of deceased donors is indisputable advantageous because of avoiding surgical risks for the donor. The main risk for a living donor is a thromboembolic event development due to the long surgical duration together with the possibility of anesthetic complications [8]. Long-term risks include ureter injury (ureteric-vaginal fistula [9] and ureteric laceration [10]). Whereas a cold ischemia time is short in living donation, longer ischemia times have to be taken into consideration when using deceased donors. Therefore, more precise knowledge about the tolerance of the uterus to prolonged cold ischemia is required in order to increase the organ donor pool and improve the general outcomes.

In separate experiments, the uterus graft was proven to be resistant to both warm and cold ischemia [11,12]. To date, several different preservation solutions, such as IGL-1^®^ [13], Celsior^®^ [14,15,16], University of Wisconsin [17,18], NaCl [17], Ringer’s acetate [18], Perfadex^®^ [18], and Custodiol^®^ [19,20,21,22,23,24,25], were used in experimental and clinical practice of UTx. Based on literature, modulation of the solution used for uterus graft static cold storage (SCS) could prevent ROS formation with resulting in reduced cell damage [17]. On the basis of Custodiol^®^ solution, the novel Custodiol-N solution was developed and supplemented with glycine and alanine to inhibit formation of hypoxia-induced plasma membrane pores and fortified with iron chelators, including deferoxamine and LK-614, to inhibit cold-induced cell injury as well as L-arginine to decrease microcirculatory disturbances [26,27]. Moreover, mannitol has been replaced by sucrose. The complete composition of the preservation solution is compiled in Table 1. In previous experiments, Custodiol-N proved to be superior to Custodiol^®^ solution concerning inhibition of hypoxic cell injury and cold-induced cell injury [26,28,29,30]. An ongoing prospective, randomized, single blind, multicenter, phase III comparison study intends to demonstrate non-inferiority of Custodiol-N against Custodiol^®^ in kidney, combined kidney-pancreas and liver transplantation (ClinicalTrials.gov Identifier: NCT03627013) [21]. Preliminary results are expected in early 2023. However, currently, no studies using the novel Custodiol-N for preserving the uterus were published.

The aim of the present study was to evaluate the effect of Custodiol-N in an experimental model of rat uterus prolonged cold preservation time compared to standard Custodiol^®^ solution. By using Custodiol-N base solution, the role of iron chelators in the composition of Custodiol-N was evaluated. Cold ischemic injury was documented by histology, including immunohistochemistry (IHC), and biochemical tissue analysis.

## 2. Results

### 2.1. Histology

**Figure 1:**
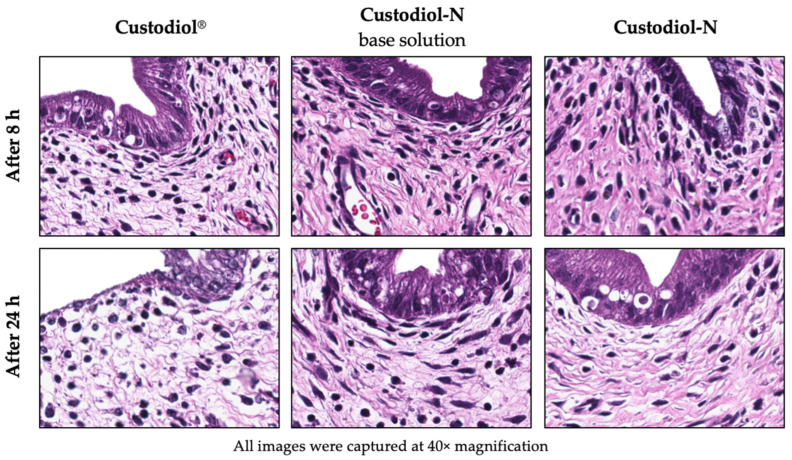
Effects of different preservation solutions on uterus SCS damage in H&E stained tissue samples.

After 8 h SCS, H&E staining revealed no differences between all three solutions (Figure 1) when comparing the total score based on our adapted scoring system for cold injury (Table 2). The median score in the Custodiol^®^ group was 2.5 (2; 3) out of 9, Custodiol-N base—2.5 (1; 3) and Custodiol-N—2 (1; 3) (*p* = 0.728). However, a significantly higher percentage of tissue edema was found in the Custodiol^®^ group when compared to Custodiol-N base and Custodiol-N (10% (5; 15) vs. 4% (0; 5) vs. 3% (3; 3), respectively, *p* = 0.004). After 8 h of SCS, the median percentage of tissue necrosis was highest in the Custodiol^®^ group when compared to the other groups, without statistical significance (*p* = 0.138). There were no significant differences observed between Custodiol-N base and Custodiol-N groups after 8 h of cold preservation.

After 24 h of SCS, the median score of the Custodiol^®^ group was highest—6 (5; 7) out of 9, Custodiol-N base—3.5 (3; 5) and Custodiol-N—3 (3; 4) (*p* = 0.008). Moreover, a significantly higher percentage of tissue edema (15% (15; 25) vs. 5% (5; 10) vs. 5% (5; 10), respectively, *p* = 0.003) and the median percentage of tissue necrosis signs (20% (10; 30) vs. 10% (5; 10) vs. 7.5% (5; 10), respectively, *p* = 0.009) were found in the Custodiol^®^ group compared to Custodiol-N base and Custodiol-N. There were no significant differences observed between Custodiol-N base and Custodiol-N groups after 24 h of SCS.

Other histological features, such as endometrial cell loss, perimetrium layer thickening, vasoconstriction, and smooth muscle contraction, were similar at all time in all three groups.

### 2.2. Tissue MPO Expression

**Figure 2:**
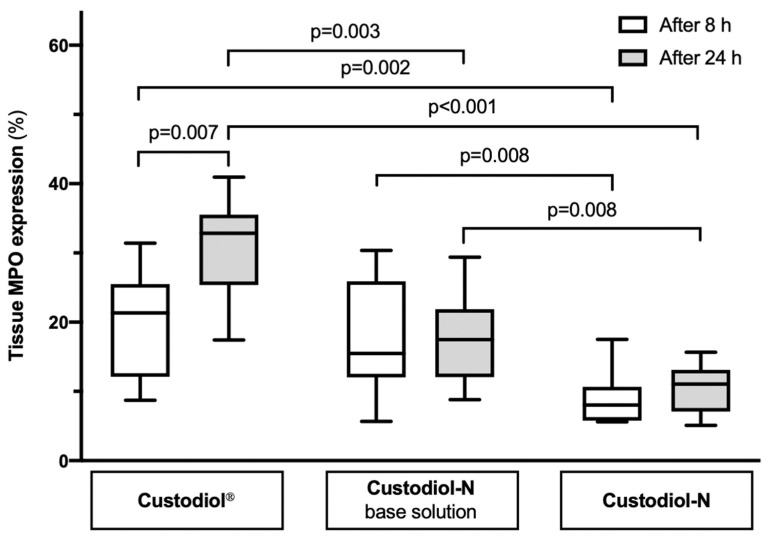
The percentage of myeloperoxidase (MPO) expression in uterus tissue cells preserved in different solutions for 8 and 24 h.

After 8 h of SCS, the median percentage of MPO expression in the Custodiol^®^ group was highest—21.34% (12.43; 25.27), followed by Custodiol-N base—15.50% (12.50; 25.31) and Custodiol-N—8.03% (5.81; 10.26) (Figure 2). Significant differences were found when Custodiol^®^ was compared to Custodiol-N (*p* = 0.002) as well as Custodiol-N base compared to Custodiol-N (*p* = 000.8). Furthermore, MPO expression increased in all three groups over time and was found to be 32.83% (27.66; 34.51) in Custodiol^®^, 17.48% (12.93; 21.11) in Custodiol-N base, and 11.07% (7.39; 12.86) in Custodiol-N group after 24 h of SCS; however, this increase was only statistically significant in the Custodiol^®^ group (*p* = 0.007). MPO expression after 24 h of SCS was significantly lower in Custodiol-N compared to Custodiol^®^ and Custodiol-N base (*p* < 0.001 and *p* = 0.008, respectively), and significantly lower in Custodiol-N base compared with Custodiol^®^ group (*p* = 0.003).

### 2.3. Tissue SOD Activity

**Figure 3:**
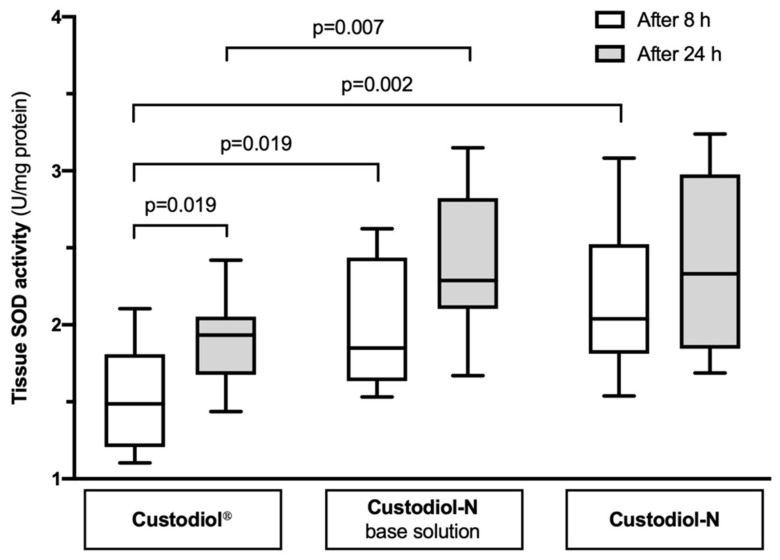
The SOD activity in uterus tissue samples preserved in different solutions for 8 and 24 h.

After 8 h of SCS, the SOD activity in uterus tissue samples was significantly increased in both Custodiol-N base (1.85 (1.66; 2.62) U/mg protein; *p* = 0.019) and Custodiol-N (2.04 (1.82; 3.08); *p* = 0.002) groups compared to the Custodiol^®^ group (1.49 (1.21; 2.10)). There was no significant difference between Custodiol-N base and Custodiol-N (*p* = 0.436) (Figure 3). Further, after 24 h of SCS, the SOD activity increased in all three groups and was found to be 1.93 (1.69; 2.42) U/mg protein in Custodiol^®^, 2.29 (2.13; 3.15) in Custodiol-N base, and 2.33 (1.86; 3.24) in Custodiol-N group. However, this increase was only statistically significant in the Custodiol^®^ group (*p* = 0.019, *p* = 0.089, *p* = 0.579, respectively). On the other hand, SOD activity after 24 h was significantly higher in Custodiol-N base (*p* = 0.007) but similar in Custodiol-N (*p* = 0.123) compared to the Custodiol^®^ group.

## 3. Discussion

In this study, the effects of Custodiol-N, a modified Custodiol^®^ solution, were investigated in a rat uterus prolonged preservation model. This novel solution in both partial (without iron chelators) and full composition achieved significant superior results over the standard Custodiol^®^ in our experiments.

In general, the formulation of Custodiol-N solution was modified in four important ways to improve the protective capacity of its predecessor Custodiol^®^ [31,32]. (1) Reduction of chloride concentration to reduce chloride-induced injury. (2) Addition of cytoprotective amino acids, such as L-arginine, glycine, and alanine. (3) Partial substitution of histidine by N-α-acetyl-L-histidine to inhibit the histidine-induced cytotoxicity. (4) Addition of the iron-chelators deferoxamine and LK-614, to reduce iron-dependent injury.

Extensive research examining cell damage after tissue SCS has shown a complex interaction of different events including production of inflammatory cytokines, increased release of reactive oxygen species (ROS), loss of epithelial integrity, microvascular damage with subsequently increased permeability, and cellular infiltration leading to cell death [33]. ROS formed in an iron-dependent manner have been discovered to play a crucial role in cold ischemia injury [32] in different cell types, including hepatocytes, endothelial, lung, and kidney cells [33,34,35]. Current research showed that addition of iron chelators to preservation solutions decreased cold preservation injury [29,33,34,35,36].

Oxygen free radical scavengers, including SOD, have been shown to protect the subcellular architecture during ischemia [37]. Significantly higher SOD activity was found in both Custodiol-N groups (with and without iron chelators) compared to Custodiol^®^ leading to reduced oxidative stress. Due to preserved antioxidative agents in ischemic cells, the generation of ROS could be reduced upon reperfusion, leading to less severe endothelial and DNA damage and local inflammatory responses [38]. Iron chelators added to Custodiol-N inhibit oxidative stress induced cell damage as well as further polymorphonuclear infiltration and tissue activation as documented by reduced MPO levels in the Custodiol-N groups.

The absence of substrate delivery during ischemia and hypothermia is known to induce Na+/K+ protein pump dysfunction leading to sodium and water retention in graft tissue [39]. The ability to counteract this effect is supposed to be one of the most important properties of preservation solutions [39,40]. In the current experiments, both Custodiol-N, with and without iron chelators, demonstrated the ability to prevent tissue edema mainly due to the fact that mannitol has been replaced with sucrose in Custodiol-N solution. Previous studies suggested that the inclusion of less permeable sugars in the preservation solution suppresses the cold ischemia-induced tissue edema more efficiently, especially during prolonged cold preservation [40,41].

Our findings are supported by previous studies that described extended SCS in experimental UTx [14,18,42]. El-Akouri et al. confirmed this tolerability by the successful pregnancies after embryo transfer to murine uterus grafts that had been preserved for 24 h [42].

The undeniable tolerance of the uterus to prolonged SCS can only be confirmed at least after reperfusion and/or pregnancy in a transplanted uterus which is the main limitation of this study. However, further research is mandatory to improve UTx results by optimization of organ preservation.

## 4. Materials and Methods

### 4.1. Animals

A total of 20 female Sprague Dawley rats (12-week-old; weight 270–360 g) were used for uterus procurement. The animals were obtained from Janvier Labs (Le Genest-Saint-Isle, France) and arrived at the research facility 7 days prior to surgery. Rats were housed four animals per cage in a controlled environment (22 ± 1 °C; 12 h/12 h light/dark cycle) and had access to fresh water and chow ad libitum. The study followed the guidelines for the handling and care of experimental animals issued by the Federation of European Laboratory Animal Science Associations (FELASA) and was approved by the Austrian Federal Ministry of Science, Research and Economy.

### 4.2. Uterus Procurement

During the procedure of uterus procurement, rats were in deep anesthesia in a supine position on a 37 °C heating pad. Anesthesia was infused by 2%, 2 L/min isoflurane applied in a rat anesthesia box. After induction therapy, rats were anesthetized with intramuscular injection of ketamine (50 mg/kg) and xylazine (9 mg/kg). A median abdominal laparotomy was performed with subsequent preparation and removal of the whole uterus. The animals were euthanized immediately afterwards by terminal blood withdrawal.

### 4.3. Experimental Groups, Static Cold Storage (SCS), and Sampling

The removed uterus horns were randomly assigned to the respective experimental groups: Custodiol^®^ (*n* = 10), Custodiol-N base (*n* = 10), and Custodiol-N (*n* = 10). Custodiol-N base solution represents Custodiol-N solution without iron chelators (Table 1). The uterus cavity was, gently without force, flushed with 100 μL respective cold preservation solution, immediately packaged in small plastic bags filled with 30 mL of cold solution, and stored on ice at 4 °C. Tissue samples were obtained after 8 and 24 h of SCS. One part of the tissue specimen was fixed in 4% formalin and embedded into paraffin blocks for histological analyses and IHC while another part was frozen and stored in liquid nitrogen for later biochemical analyses.

### 4.4. Histology

Tissue sections (2 μm thick) were stained with hematoxylin and eosin (H&E) and subsequently examined under a light microscope by a blinded, experienced pathologist. For histological evaluation, a semi-quantitative morphological scoring system was modified based on previously published methods [43,44] (Table 2).

### 4.5. Immunohistochemical (IHC) Staining

Expression levels of the oxidative stress marker myeloperoxidase (MPO) in uterus tissue was assessed by IHC. Anti-MPO (Dako, Via Real Carpinteria, CA, USA; dilution 1:800; polyclonal rabbit anti-human) antibodies were used in combination with the UltraVision LP Detection System HRP Polymer (Thermo Fisher Scientific, Waltham, MA, USA) and DAB chromogen (Dako, Via Real Carpinteria, CA, USA). For positive control, rat spleen tissue was used, while for negative control, primary antibodies were omitted. Stained slides were scanned and analyzed using the QuPath software version 0.2.0-m5 (Belfast, Northern Ireland) [45]. The number of positive cells was counted in a blinded fashion and expressed as percentage stained cells of total nuclei in the entire tissue comprising all uterus layers.

### 4.6. Biochemistry

For biochemical analyses, frozen tissue samples were homogenized in ice cold phosphate-buffered saline using a bead-beater, MagNA Lyser (at 6500 rpm/30 s × 3). To prevent excess sample peroxidation while processing, 5 mM butylated hydroxytoluene (antioxidant) was added in advance. The supernatant was collected, aliquoted, and stored at −80 °C for later analyses. Superoxide dismutase (SOD) activity was determined using the commercially available SOD Colorimetric Activity Kit produced by Thermo Fisher Scientific (Waltham, MA, USA) exactly as described by the manufacturer. Results were adjusted to total protein levels determined by the BCA Protein Assay Kit (Thermo Fisher Scientific, Waltham, MA, USA) and expressed as units per mg of protein.

### 4.7. Statistical Analyses

Statistical analyses were performed using SPSS (Statistical Package for the Social Sciences) version 23.0 (IBM Corp., Armonk, NY, USA). Kruskal–Wallis and Mann–Whitney U tests were used to analyze statistical difference between groups according to their distribution. Non-parametric data is presented as median and quartiles (Q1; Q3). A *p* value less than 0.05 was considered statistically significant.

## 5. Conclusions

This study demonstrates the superiority of Custodiol-N solutions for uterus graft preservation when compared to standard Custodiol^®^ most likely via inhibition of oxidative stress and tissue edema. It seems that iron chelators included in Custodiol-N play an important protective role against cold ischemic injury. The effects of this novel preservation solution are promising and further research is warranted.

## Figures and Tables

**Table 1:** Composition of the clinically used Custodiol^®^ and Custodiol-N solution.

Components (mmol/L)	Custodiol^®^	Custodiol-N Base Solution	Custodiol-N
Sodium	16	16	16
Potassium	10	10	10
Magnesium	4	8	8
Calcium	0.015	0.02	0.02
Chloride	50	30	30
L-Histidine	198	124	124
N-α-acetyl-L-Histidine	–	57	57
Aspartate	1	5	5
Tryptophan	2	2	2
α-Ketoglutarate	2	2	2
Arginine	–	3	3
Alanine	–	5	5
Sucrose	–	33	33
Mannitol	30	–	–
Glycine	–	10	10
Deferoxamine	–	–	0.025
LK-615	–	–	0.0075

Data modified from Kniepeiss et al. [26]. Custodiol-N base solution is Custodiol-N without iron chelators, deferoxamine, and LK-615.

**Table 2:** The scoring system for uterus static cold storage (SCS) damage evaluation.

Histological Findings	Score			
	0	1	2	3
Edema	<5%	<5–15%	15–30%	>30%
Necrosis	Absent	<15%	15–30%	>30%
Smooth muscle contraction	Absent	Present		
Impaired basement membrane integrity	Absent	Present		
Endometrial cells loss	Absent	Present		

The maximum score for uterus SCS injury is 9. Percentages are calculated as (surface of the affected area/surface of the whole section) × 100.

## Abbreviations

AUFI	absolute uterine factor infertility
UTx	uterus transplantation
H&E	hematoxylin and eosin
IHC	immunohistochemistry
IRI	ischemia/reperfusion injury
MPO	myeloperoxidase
ROS	reactive oxygen species
SCS	static cold storage
SOD	superoxide dismutase
